# Garlic: An Alternative Treatment for Group B Streptococcus

**DOI:** 10.1128/Spectrum.00170-21

**Published:** 2021-11-24

**Authors:** Kátia Andrea de Menezes Torres, Sônia Maria Rolim Rosa Lima, Luce Maria Brandão Torres, Maria Thereza Gamberini, Pedro Ismael da Silva Junior

**Affiliations:** a Post-Graduation Program, Santa Casa de Sao Paulo of Medical Sciences, São Paulo, Brazil; b Department of Obstetrics and Gynecology, Santa Casa de Sao Paulo School of Medical Sciences, São Paulo, Brazil; c Environmental Reaserch Institute, Department of Infrastructure and Environment, São Paulo, Brazil; d Department of Physiological Sciences, Santa Casa de Sao Paulo School of Medical Sciences, São Paulo, Brazil; e Laboratory for Applied Toxinology, Center of Toxins, Immune-Response and Cell Signaling (CeTICS/CEPID), Butantan Institute, São Paulo, Brazil; University of Illinois at Urbana Champaign

**Keywords:** *Allium sativum* L., *Streptococcus agalactiae*, Ƴ-glutamyl-*S*-allyl-cysteine, Ƴ-glutamyl-phenylalanine, ajoene, antimicrobial, antimicrobial activity, bacterial growth kinetics, organosulfur compounds

## Abstract

Prenatal screening in pregnant women between 35 and 37 weeks of gestation and intrapartum antibiotic prophylaxis has successfully reduced the incidence of neonatal morbidity and mortality related to Streptococcus agalactiae. However, the contamination rates of newborns are still considerable. In traditional and folk medicines, it has been observed that garlic has been effective in treating S. agalactiae infection. The aim of this study was to isolate and identify the active compounds from garlic that have antimicrobial activity against S. agalactiae. In order to do this, SP80 (Sep-Pak 80%) obtained from crude garlic extract (CGE) was fractionated by reverse-phase ultrafast liquid chromatography with UV (RP-UFLC-UV) using a Shim-pack PREP-ODS column. All fractions obtained were tested using a microbial growth inhibition test against the S. agalactiae strain (ATCC 12386). Five clinical isolates were used to confirm the action of the fractions with antimicrobial activity, and the bacterial growth curve was determined. Identification of the antimicrobial compounds was carried out through liquid chromatography coupled with mass spectrometry (LC/MS) and nuclear magnetic resonance (NMR). The active compounds found to exhibit antimicrobial activity were Ƴ-glutamyl-*S*-allyl-cysteine (fraction 18), Ƴ-glutamyl-phenylalanine (fraction 20), and the two stereoisomers (*E* and *Z*) of ajoene (fraction 42). The MICs of these fractions were 5.41 mg/ml, 4.60 mg/ml, and 0.16 mg/ml, respectively, and they inhibited the growth of the clinical isolates tested. Antimicrobial compounds from garlic may be a promising source in the search for new drugs against S. agalactiae.

**IMPORTANCE** Invasive disease due to group B streptococcal (GBS) infection results in a wide spectrum of clinical disease in neonates. Maternal colonization by GBS is the primary risk factor for disease. The strategy recommended by the Centers for Disease Control to reduce neonatal GBS infection is the culture-based screening of all pregnant women at 35 to 37 weeks of gestation and intrapartum antibiotic prophylaxis (IAP). However, indiscriminate use of antibiotics favors the selection and spread of resistant bacteria. The global scenario of antibacterial resistance has been of great concern for public health, and natural products can be a source of new substances to help us grapple with this problem.

## INTRODUCTION

Streptococcus agalactiae, also known as group B Streptococcus (GBS) is a round Gram-positive coccus bacterium which inhabits the gastrointestinal and genitourinary tracts of humans as a commensal organism (1). In the 1970s, studies showed the pathogenicity of this microorganism, associating it with bacteremia, pneumonia, and meningitis in infants under 3 months of age (2).

Neonatal GBS disease is an invasive infection affecting newborns during the first weeks of life and is a major cause of morbidity and mortality during this period. It can be divided into two types. The first type is early-onset GBS disease (EOGBS), which manifests within the first 72 h of life and can be acquired through vertical transmission from mother to fetus before and during childbirth. Its symptoms include respiratory disease, sepsis, and meningitis. The second type is late-onset GBS disease (LOGBS), which emerges between the 4th and 90th days of life and is acquired through contact of the newborn with the environment, presenting with symptoms such as sepsis, meningitis, urinary tract infection, osteoarthritis, respiratory disease, and cellulitis (3).

In 1996, prophylactic recommendations by the American College of Obstetricians and Gynecologists (ACOG) (4) and the Centers for Disease Control and Prevention (CDC) (5) were implemented to prevent GBS infections in newborns, and in 1997, the American Academy of Pediatrics (AAP) issued its own guidelines (6). These included measures such as the use of antibiotics during childbirth.

In 2010, the CDC published a revised consensus opinion, recommending vaginal cultures for all pregnant women between 35 and 37 weeks of gestation, with intrapartum antibiotic prophylaxis (IAP) being given to at-risk women (7).

In the absence of IAP, around 50% of newborns of mothers with GBS positive cultures are colonized by S. agalactiae, and 1 to 2% can develop EOGBS disease (2). Although prophylactic measures are well established in conventional medicine, herbal-based medicines are widely used for disease prevention in developing countries such as Brazil, and there is a history in traditional medicine of the use of garlic for the treatment of GBS infection in pregnant women (8, 9).

Garlic (Allium sativum L.), part of the family Liliaceae, besides being widely used as a food and condiment, has a series of therapeutic properties attributed to it in traditional medicine (10), many of which have yet to be scientifically confirmed. This plant species has been described as having antioxidant (11), antitumoral (12), anti-inflammatory (13), immunomodulatory (14), antiviral (15), antimicrobial (16, 17), and cardiovascular protective (18) actions, as well as promoting beneficial effects in diabetic (19) and obese patients (20).

Organosulfur compounds are the main bioactive compounds present in garlic, with allicin, which is responsible for garlic’s strong odor, being the most commonly described compound with medicinal activity in the literature (21).

Allicin was identified by Cavallito and Bailey (22) and has been demonstrated to have antimicrobial activities against Gram-positive and Gram-negative bacteria (23). It is converted from its precursor alliin by the enzyme alliinase when tissue damage occurs. It is quite unstable and quickly participates in a cascade of nonenzymatic reactions to produce compounds such as vinyldithiins, ajoenes, and (poly)sulfides, which have been reported to exhibit antimicrobial activity (21, 24).

Ajoenes (*E* and *Z*) are also sulfur-containing compounds, and their antimicrobial activity has been demonstrated against Gram-positive and negative bacteria and fungi, such as Aspergillus niger and Candida albicans (24).

In view of the ethnopharmacological information and antimicrobial properties described to date, the objective of the present study was to assess the actions of garlic and its active principals isolated from the plant against S. agalactiae bacterium. The study sought to assess information gleaned from traditional medicine and isolate the chemical constituents with pharmacological activity, thereby expanding the armamentarium of treatment options available for the management of infections by the bacterium in pregnant women.

## RESULTS

### Isolation/purification.

The antimicrobial activity of *A. sativum* L. against S. agalactiae strain ATCC 12386 was investigated, revealing that incubation of crude garlic extract (CGE) on culture plates inhibited the bacterial growth. It was, therefore, then submitted to a solid-phase extraction (SPE) on Sep-Pak C_18_ cartridges, using 80% acetonitrile (ACN) + 0.05% trifluoroacetic acid (TFA) solution as the mobile phase. This step provided the SP80 fraction, which maintained antimicrobial activity against S. agalactiae ATCC 12386.

**FIG 1:**
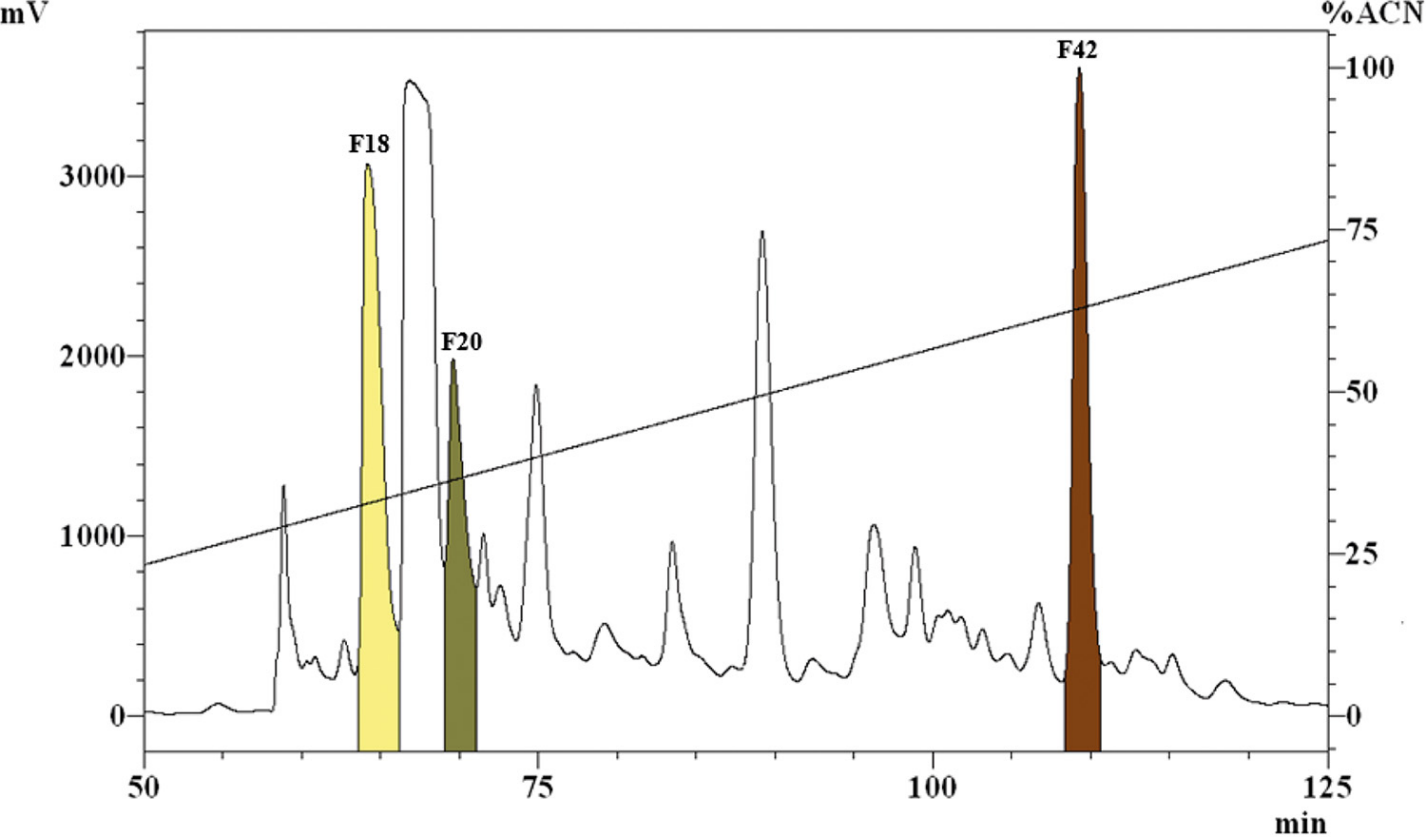
Chromatographic profile of SP80, obtained from Sep-Pak C_18_ cartridges. The analysis was performed using an RP-UFLC system (Shimadzu Prominence device) with a preparative reverse-phase column, Shim-pack PREP-ODS (250 mml × 50 mm inside diameter [i.d.], 15 μm), and the absorbance was monitored at 225 nm. Note the peaks for fractions 18, 20, and 42 (F18, F20, and F42, respectively) that correspond to the fractions with antimicrobial activity against S. agalactiae ATCC 12386.

Reverse-phase ultrafast liquid chromatography with UV spectroscopy (RP-UFLC-UV) of the SP80 provided fractions according to retention time (Rt) and polarity. Biomonitoring of these fractions (46 in total) disclosed antimicrobial activity in only fractions 18, 20, and 42 (Fig. 1). The yields of these fractions were 8.48%, 2.77%, and 0.31%, respectively.

### Liquid growth inhibition assay and MIC.

CGE, SP80, and all fractions (25) were evaluated for antimicrobial activity against S. agalactiae ATCC 12386, and the microbial growth was measured by monitoring the increase in optical density at 595 nm (OD_595_) using a Victor3 1420 multilabel counter plate reader (Perkin Elmer, Waltham, MA, USA). The values are shown in Table 1. The MICs against S. agalactiae ATCC 12386 were determined for the fractions with antimicrobial activity (fractions 18, 20, and 42) and for CGE and SP80. These results are shown in Table 2 and are compared with the MIC of penicillin G as the positive control in the liquid growth inhibition assay. The results of these microbiological assays showed that the greatest growth inhibition was achieved when the bacteria of S. agalactiae ATCC 12386 were incubated with fraction 42.

**TABLE 1:** Results of growth inhibition determined by measuring the absorbance at 595 nm using a Victor3 1420 instrument (Perkin Elmer) against S. agalactiae strain ATCC 12386 of all fractions obtained from bio-guided fractionation of SP80^*a*^

Fraction	OD
F1	0.239
F2	0.240
F3	0.225
F4	0.240
F5	0.211
F6	0.239
F7	0.234
F8	0.221
F9	0.211
F10	0.202
F11	0.262
F12	0.259
F13	0.258
F14	0.233
F15	0.250
F16	0.228
F17	0.213
**F18**	**0.036**
F19	0.240
**F20**	**0.040**
F21	0.247
F22	0.247
F23	0.228
F24	0.255
F25	0.229
F26	0.227
F27	0.224
F28	0.220
F29	0.219
F30	0.214
F31	0.241
F32	0.243
F33	0.227
F34	0.213
F35	0.218
F36	0.236
F37	0.242
F38	0.210
F39	0.220
F40	0.214
F41	0.243
**F42**	**0.038**
F43	0.236
F44	0.216
F45	0.253
F46	0.221
**CGE** ^*b*^	**0.038**
**SP80^*c*^**	**0.037**

a Positive control (penicillin G): 0.036 optical density (OD; absorbance); negative control (sterile water and TSB), 0.215 OD. Fractions in bold are those that showed antimicrobial activity.

b CGE (crude garlic extract).

c SP80 were obtained after solid-phase extraction was performed using Sep-Pak C_18_ cartridge, with 80% ACN + 0.05% TFA solution as mobile phase. F18, 20 and 42 fraction with antimicrobial activity.

**TABLE 2:** Observed MIC for CGE, SP80, and antimicrobial fractions against Streptococcus agalactiae ATCC 12386

*Allium sativum* L.	MIC^*a*^ (mg/ml)	SD
CGE^*b*^	8.75	0.04
SP80^*c*^	2.40	0.42
F18	5.41	0.79
F20	4.60	0.69
F42	0.16	0.02
Penicillin G^*d*^	0.038 μg/ml	0.01

a MIC was performed in triplicate and expressed as mean ± standard deviation.

b CGE, crude garlic extract.

c The SP80 sample was obtained after solid-phase extraction was performed using a Sep-Pak C_18_ cartridge, with 80% ACN + 0.05% TFA solution as the mobile phase.

d Penicillin G, first-choice antibiotic for prophylaxis against S. agalactiae infection.

Strains of clinical isolates of S. agalactiae provided by the Salomão Zoppi, Sao Paulo, SP, Brazil, were used in the evaluation of the antimicrobial activity of fractions 18, 20, and 42 against S. agalactiae. Using the MICs determined for S. agalactiae ATCC 12386, all fractions inhibited the growth of the clinical isolates tested.

### Bacterial growth curve kinetics.

**FIG 2:**
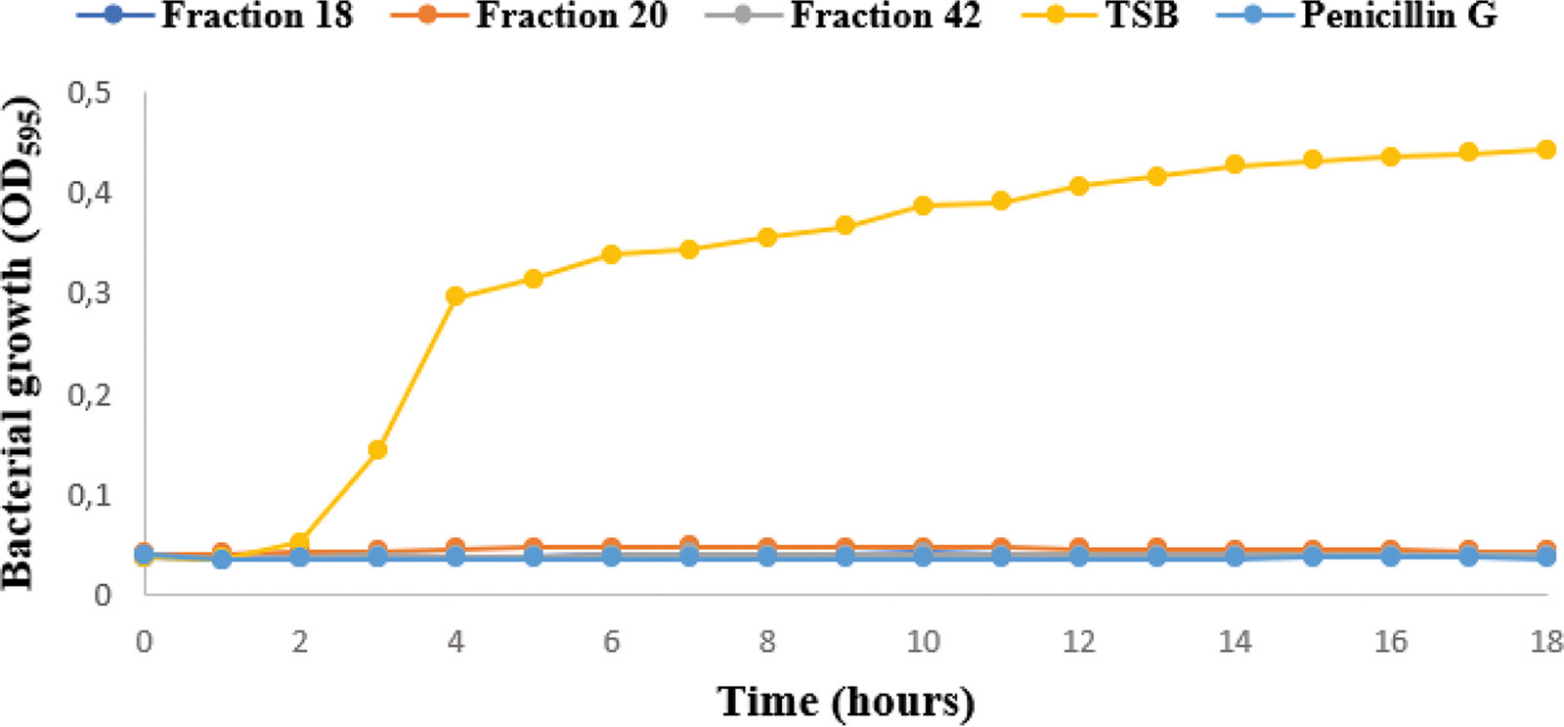
Bacterial growth curve for Streptococcus agalactiae ATCC 12386 in the presence of fractions 18, 20, and 42, in tryptic soy broth (TSB) as the negative control.

Investigation of the bacterial growth kinetics in TSB medium was conducted to evaluate the antibacterial potency of fractions 18, 20, and 42 against S. agalactiae ATCC 12386. Figure 2 shows the optical density at 595 nm for the different incubation times. After 18 h of incubation, the negative-control (TSB) growth curve achieved exponential growth, while the fractions and penicillin G (positive control) showed no growth. The fractions and positive and negative controls were plated onto blood agar plates, and only the negative control showed bacterial growth.

### Mass spectrometry (LC-MS) and NMR.

**FIG 3:**
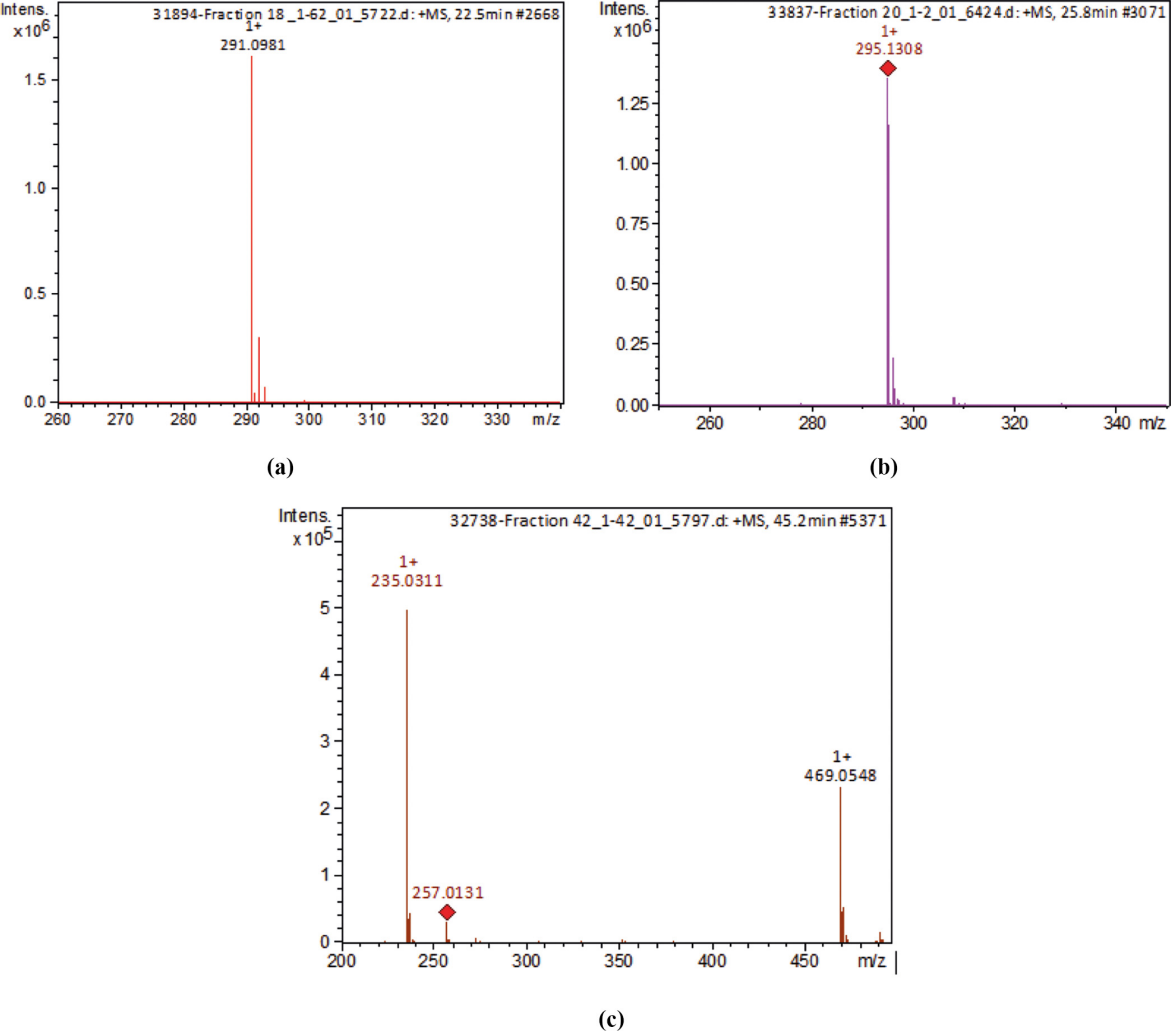
Mass spectrometry (MS) using a Shimadzu model CBM-20A chromatograph. (a) Mass spectrum for Ƴ-glutamyl-*S*-allyl-cysteine (GSAC) (fraction 18); (b) mass spectrum for Ƴ-glutamyl-phenylalanine (fraction 20); and (c) mass spectrum for *E*- and *Z*-ajoenes (fraction 42).

After confirmation of the antimicrobial activity of fractions 18, 20, and 42, phytochemical analyses were performed to identify the bioactive compounds present in these fractions. The data for fraction 18 were detected using liquid chromatography-diode array detection (LC-DAD) at 225 nm and a retention time (Rt) of 22.5 min in the total ion chromatogram (TIC) and liquid chromatography coupled with mass spectrometry (LC-MS) for a molecular ion peak of [M+H]^+^ = 291.0981 Da (Fig. 3a). The data for fraction 20 were detected using LC-DAD at 225 nm and an Rt of 25.8 min in the TIC and LC-MS for a molecular ion peak of [M+H]^+^ = 295.1308 Da (Fig. 3b). The data for fraction 42 were detected using LC-DAD at 225 nm and an Rt of 45.2 min in the TIC and LC-MS for molecular ion peaks of [M+H]^+^ = 235.0311 Da, [M+Na]^+^ = 257.0131, and [2M+H]^+^ = 469.0548 Da (Fig. 3c).

**FIG 4:**
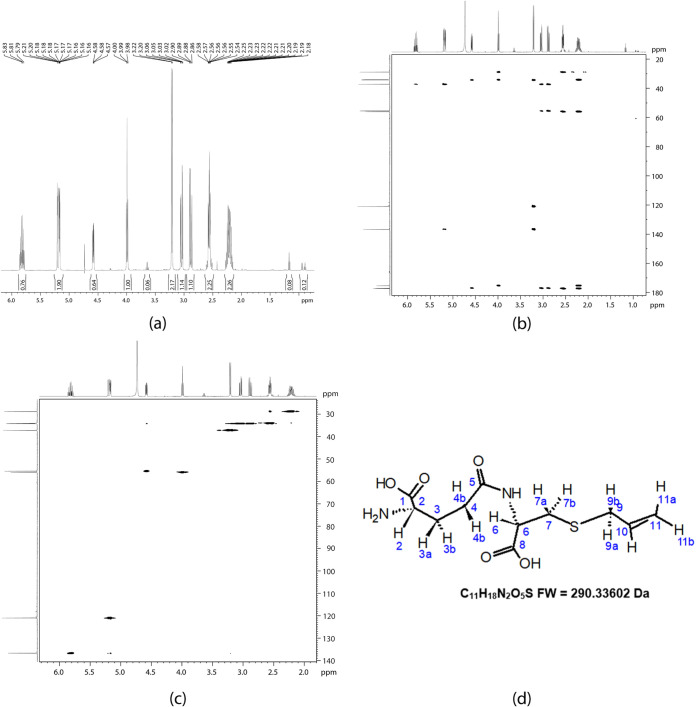
Nuclear magnetic resonance (NMR) spectra using a Bruker Daltonics maXis 3G instrument (500 MHz) in deuterated water (D_2_O): (a) 1D ^1^H NMR spectrum showing the multiplicity and the coupling constants; (b) 2D HMBC spectrum, providing correlations between carbons that are mainly separated by two or three bonds; (c) 2D HSQC spectrum—several dots are present, with each one representing a correlation between ^1^H and ^13^C; and (d) chemical structure for Ƴ-glutamyl-*S*-allyl-cysteine (fraction 18). FW, formula weight.

**FIG 5:**
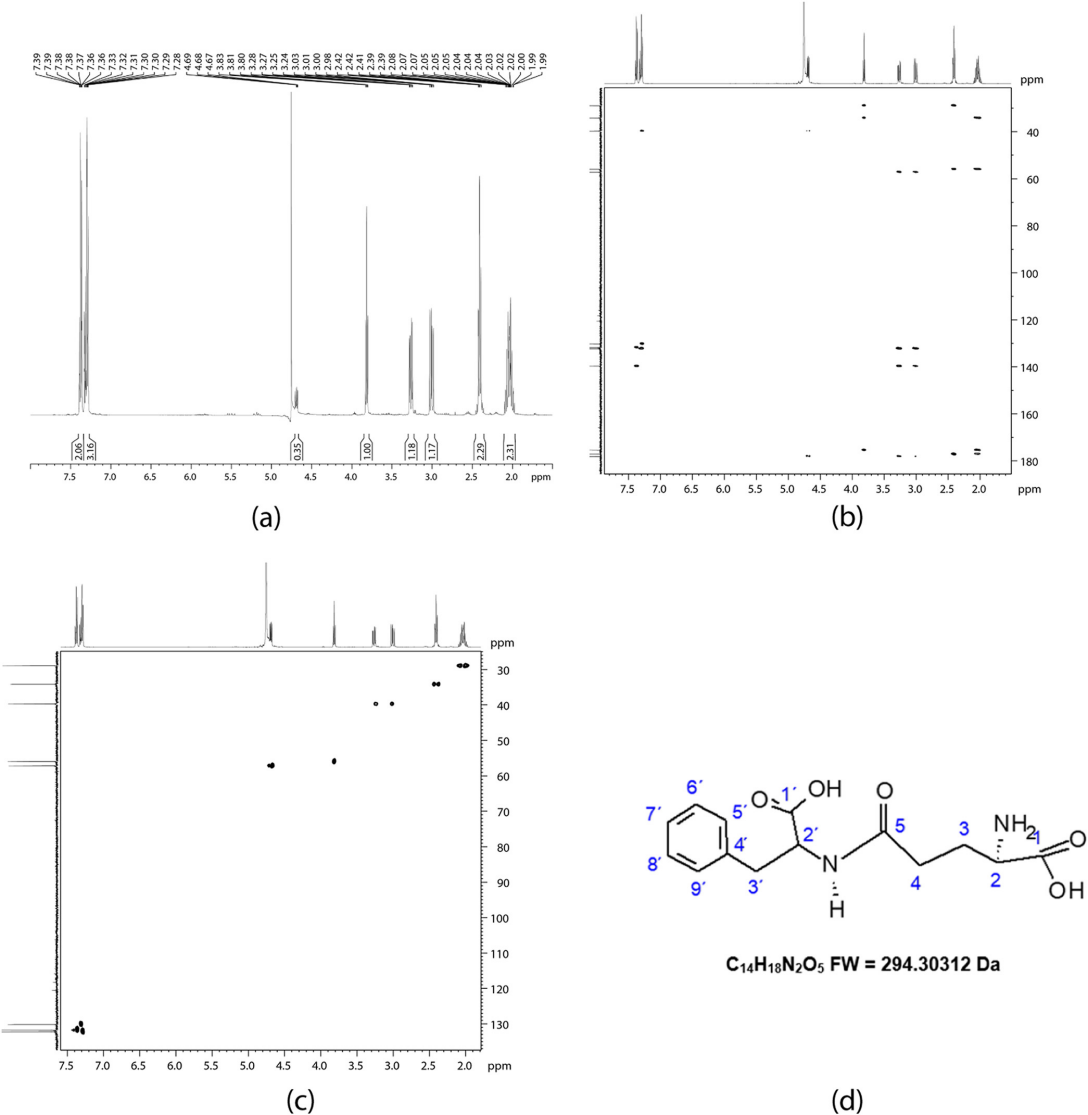
Nuclear magnetic resonance (NMR) spectra (Bruker Daltonics maXis 3G instrument, 500 MHz) in deuterated water (D_2_O): (a) 1D ^1^H NMR spectrum showing the multiplicity and the coupling constants; (b) 2D HMBC spectrum, providing correlations between carbons that are mainly separated by two or three bonds; (c) 2D HSQC spectrum—several dots are present, with each one representing a correlation between ^1^H and ^13^C; and (d) chemical structure for Ƴ-glutamyl-phenylalanine (fraction 20). FW, formula weight.

**FIG 6:**
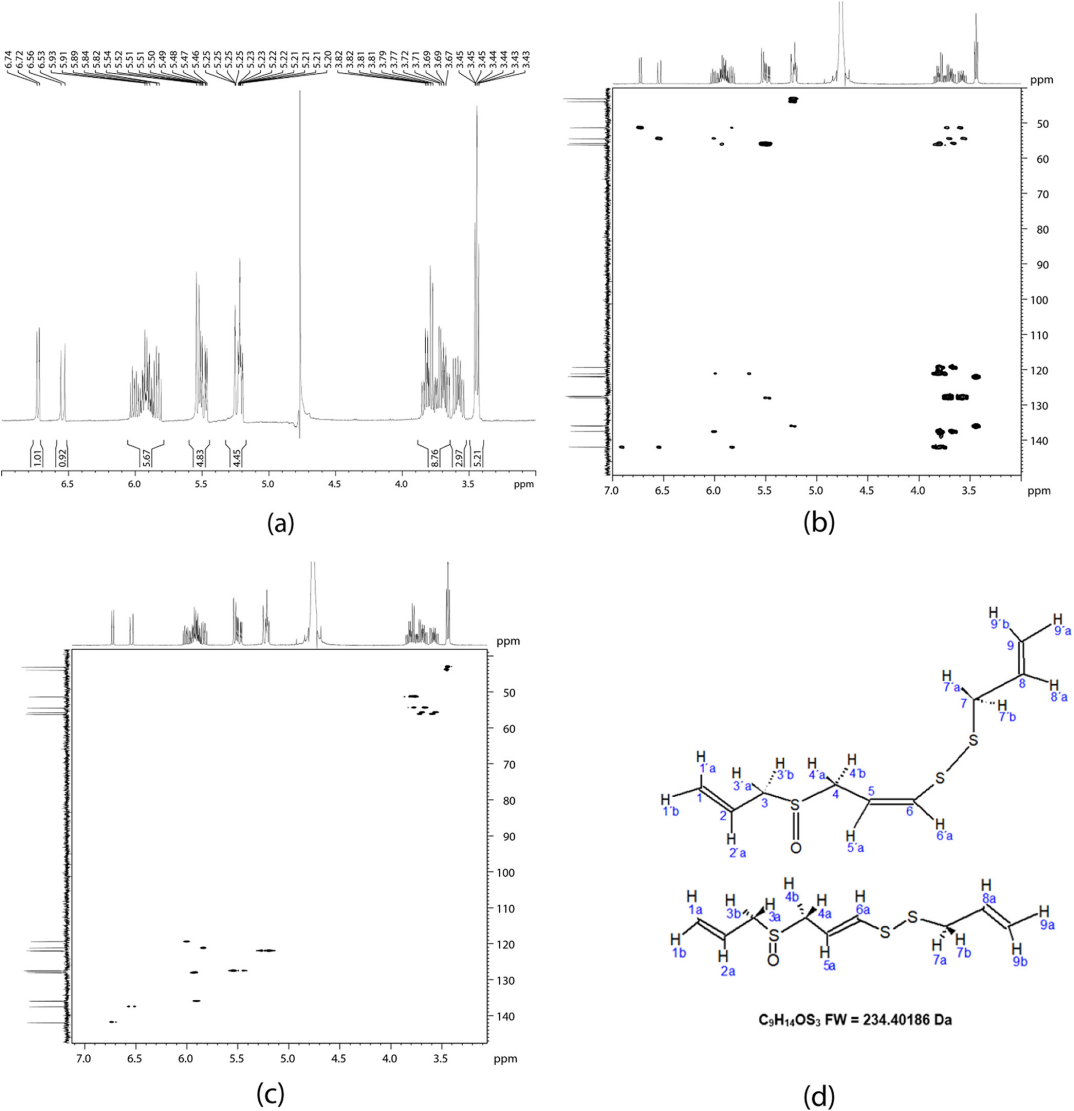
Nuclear magnetic resonance (NMR) spectra (Bruker Daltonics maXis 3G instrument, 500 MHz) in deuterated water (D_2_O): (a) 1D ^1^H NMR spectrum showing the multiplicity and the coupling constants; (b) 2D HMBC spectrum, providing correlations between carbons that are mainly separated by two or three bonds; (c) 2D HSQC spectrum—several dots are present, with each one representing a correlation between ^1^H and ^13^C; and (d) chemical structure for *Z*-ajoene (top) and *E*-ajoene (bottom) (fraction 42). FW, formula weight.

These data were confirmed by nuclear magnetic resonance (NMR). The results showed that fraction 18 corresponded to Ƴ-glutamyl-*S*-allyl-cysteine (GSAC) (Fig. 4), fraction 20 to Ƴ-glutamyl-phenylalanine (Fig. 5), and fraction 42 corresponded to the ajoenes *E* and *Z* (Fig. 6); these results were corroborated using the PubChem database (26, 27) (Tables 3, 4, and 5).

**TABLE 3:** Uni- and bidimensional NMR in deuterated water (D_2_O) for fraction 18 (Ƴ-glutamyl-*S*-allyl-cysteine), isolated from garlic^*a*^

Carbon no.	Functional group	^1^H (δ) or ppm	m, *J* = Hz^*b*^	HSQC (^13^C) δ	HMBC δ ^1^H δ (*J*^3^, *J*^2^)
1	C=O			175.3	2.2 ^1^H_3a,b_ (*J*^3^)
					2.6 ^1^H_4a,b_ (*J*^3^)
					3.9 ^1^H_2_ (*J*^2^)
2	CH	3.9 H_2_	t, *J* = 6.45 Hz	55.8	2.2 ^1^H_3a,b_ (*J*^2^)
					2.6 ^1^H_4a,b_ (*J*^3^)
3	CH_2_	2.2 H_3a_, H_3b_	m (dq)	28.9	2.6 ^1^H_4a,b_ (*J*^2^)
					3.9 ^1^H_2_ (*J*^2^)
4	CH_2_	2.6 H_4a_, H_4b_	m (dt)	34.0	2.2 ^1^H_3a,b_ (*J*^2^)
					3.9 ^1^H_2_ (*J*^3^)
5	C=O			170.0	2.2 ^1^H_3a,b_ (*J*^3^)
					4.6 ^1^H_6_ (*J*^3^)
6	C_6_H (NH)-CH	4.6 H_6_	dd *J* = 4.8 and 4.6 Hz	55.4	3.05 ^1^H_7a,7b_ (*J*^2^)
					3.29 ^1^H_9a,b_ (*J*^3^)
7	CH_2_	2.87 H_7a_	dd *J* = 3.05 H_7a_ *J* = 4.8 Hz and 4.8 Hz and *J* = 13.5 Hz	34.2	3.29 ^1^H_9a,b_ (*J*^3^)
					4.6 ^1^H_6_ (*J*^2^)
		3.05 H_7b_	^1^H-C_7a_ = 2.87 ppm. dd *J* = 8.20 and 13.5 Hz		
8	C=O			177.1	2.87 ^1^H_7a_ (*J*^3^)
					3.05 ^1^H_7b_ (*J*^3^)
9	CH_2_-S	3.29 (H_9a_ and H_9b_)	d, *J* = 7.20 Hz	37.2	2.87 ^1^H_7a_ (*J*^3^); 3.05 ^1^H_7b_ (*J*^3^); 5.20 (*J*^3^)^1^H_11a (_*_trans_*_)_, 5.17^1^H_11b (cis)_, and 5.83 ^1^H_10_
10	(CH=)	5.83 H_10_	dq	136.8	3.29 ^1^H_9a,b_; 5.20 ^1^H_11a (_*_trans_*_)_
					5.17 ^1^H_11b (_*_cis_*_)_
11		5.20 H_11a (_*_trans_*_)_	C_11_–H_11b (_*_geminal_*_)_ and H_11a (_*_transvicinal_*_)_	121.0	3.29 ^1^H_9a,b_ (*J*^3^)
		5.17 H_11b (_*_cis_*_)_			

a NMR performed using a Bruker Daltonics maXis 3G instrument, 500 MHz.

b m, multiplet; dq, doublet of quartets; dt, doublet of triplets; dd, doublet of doublets; d, doublet.

**TABLE 4:** Uni- and bidimensional NMR in deuterated water (D_2_O) for fraction 20 (Ƴ-glutamyl-phenylalanine), isolated from garlic^*a*^

Carbon no.	Functional group	^1^H (δ) or ppm	m, *J* = Hz^*b*^	HSQC (^13^C) δ	HMBC δ ^1^H δ (*J*^3^, *J*^2^)
1	C=O			175.5	2.06 ^1^H_3a,b_ (*J*^3^)
					3.80 ^1^H_2b_ (*J*^2^)
2	CH	3.80 H_2_	3.82 d, *J* = 10 Hz	56.0	2.06 ^1^H_3a,b_ (*J*^2^)
			3.80 d, *J* = 5 Hz		2.48 ^1^H_4a,b_ (*J*^3^)
3	CH_2_	2.06 H_3a,b_	dq 10 Hz	29.0	3.80 ^1^H_2b_ (*J*^2^)
					2.48 ^1^H_4a,b_ (*J*^2^)
4	CH_2_	2.48 H_4a,b_	dq	34.0	3.80 ^1^H_2b_ (*J*^3^)
					2.06 ^1^H_3a,b_ (*J*^2^)
5	C=O			177.0	2.02 ^1^H_3a,b_ (*J*^3^)
					2.41 ^1^H_4a,b_ (*J*^3^) (*J*^2^)
1′	C=O			179.0	2.02 ^1^H_3′a_^1^H_3′b'_
					4.68 ^1^H_2′b_ dd (*J*^2^)
2′	CH	4.68 H_2′b_		57.0	3.00 ^1^H_3′a_ (*J*^2^)
					3.25 ^1^H_3′b_ (*J*^2^)
3′	CH_2_	3.00 H_3′a_ and 3.25 H_3′b_	dd, *J* = 5 Hz	40.0	4.68 ^1^H_2′b_ dd (*J*^2^)
					7.28 H_5′_
4′	CH*_aromatic_*			140.0	3.00 ^1^H_3′a_ 3.25 ^1^H_3′b_ (*J*^3^)
					4.68 ^1^H_2′b_ (*J*^3^)
5′ and 9′	CH*_aromatic_*	7.28 H_5′_ and H_9_	m	132.0	3.00 ^1^H_3′a_ (*J*^2^)
					3.25 ^1^H_3′b_ (*J*^2^)
6′ and 8′	CH*_aromatic_*	7.36	m	131.9	3.00 ^1^H_3′a_ (*J*^3^)
					3.25 ^1^H_3′b_ (*J*^3^)
7′	CH*_aromatic_*	7.30 H_5′_ and H_9_	m	130.0	7.28 ^1^H_5′_ and H_9′_ (*J*^3^)

a NMR performed using a Bruker Daltonics maXis 3G instrument, 500 MHz.

b m, multiplet; dq, doublet of quartets; dt, doublet of triplets; dd, doublet of doublets; d, doublet.

**TABLE 5:** Uni- and bidimensional NMR in deuterated water (D_2_O) for fraction 42 (*E*- and *Z*-ajoenes), isolated from garlic^*a*^

Data for *E*-ajoene:						Data for *Z*-ajoene:					
Carbon no.	Functional group	^1^H (δ) or ppm	m, *J* = Hz^*b*^	HSQC (^13^C) δ	HMBC ^1^H δ (*J*^3^, *J*^2^)	Carbon no.	Functional group	^1^H (δ) or ppm	m, *J* = Hz^*b*^	HSQC (^13^C) δ	HMBC ^1^H δ (*J*^3^, *J*^2^)
1	CH_2_=	5.53 H_1a_ and 5.48 H_1b_	d, *J* = 10 Hz	127.0	3.60, 3.73 H_3a,b_ (*J*^3^)	1′	CH_2_=	5.53 H_1′a_ and 5.48 H_1′b_	d, *J* = 10 Hz	127.0	3.56, 3.70
	CH=		dq, *J* = 15Hz, 10 Hz, and 5 Hz				CH=		dq, *J* = 15 Hz, 10 Hz, 5 Hz		H_3′a,b_ (*J*^3^)
2		5.90 H_2a_	m	127.5	3.60, 3.73 H_3a,b_ (*J*^2^)	2′		5.90 H_2′a_	m	127.5	3.56, 3.70 H_3a,b_ (*J*^2^)
					5.53 H_1_						5.48 H_1′_
3	CH_2_=	3.60 H_3a_ and 3.73 H_3b_	dq, *J* = 10 Hz	56.2	5.53, 5.48 H_1a,b_ (*J*^3^)	3′	CH_2_=	3.56 H_3′a_ and 3.70 H_3′b_	dq, *J* = 5 Hz, 10 Hz	55.73	5.53, 5.48 H_1′a,b_ (*J*^3^)
	CH_2_=		dq, *J* = 5 Hz, 10 Hz		5.90 H_2_ (*J*^2^), 3.65, 3.83 H_4a,b_ (*J*^2^)		CH_2_=		dq, *J* = 5 Hz, 10 Hz		3.78, 3.80 H_4′a,b_
4	CH=	3.65 H_4a_ and 3.83 H_4b_	dq, *J* = 10 Hz, 5 Hz	51.5	6.54 H_6a_ (*J*^3^); 6.0 H_5a_ (*J*^2^)	4′	CH=	3.78 H_4′a_ and 3.80 H_4′b_	dq, *J* = 10 Hz, 5 Hz, 18 Hz	51.3	6.73 H_6′a_ (*J*^3^)
	CH=				3.60, 3.73 H_3a,b_ (*J*^2^)		CH=				5.83 H_5′a_ (*J*^2^)
											3.56, 3.70 H_3a,b_
5		5.83 H_5a_	dq, *J* = 5 Hz, *J* = 15 Hz	121.0	3.65, 3.83 H_4a,b_ (*J*^2^)	5′		6.00 H_5′a_	dq, *J* = 10 Hz	119.0	3.78, 3.80 H_4′a,b_ (*J*^2^)
6		6.54 H_6a_	d, *J* = 15 Hz	137.5	3.65, 3.83 H_4a,b_ (*J*^3^)	6′		6.73 H_6′a_	d, *J* = 10 Hz	142.0	3.78, 3.80 H_4′a,b_ (*J*^3^)
7	CH_2_=	3.43 H_7a,b_	d, *J* = 5 Hz	43.0	5.20, 5.25 H_9a,b_ (*J*^3^)	7′	CH_2_=	3.45 H_7′a,b_	d, *J* = 5 Hz	44.0	5.20, 5.25 H_9′a,b_ (*J*^3^)
	CH=						CH=				
8		5.95 H_8a_	m	136.1	3.43 H_7a,b_ (*J*^2^)	8′		5.95 H_8′a_	m	136.0	3.45 H_7′a,b_ (*J*^2^)
9	CH_2_=	5.20 H_9a,b_	d, *J* = 5 Hz	122.1	3.43 H_7a,b_ (*J*^3^)	9′	CH_2_=	5.25 H_9′a,b_	d, *J* = 5 Hz	122.0	3.45 H_7′a,b_ (*J*^3^)

a NMR performed using a Bruker Daltonics maXis 3G instrument, 500 MHz.

b m, multiplet; dq, doublet of quartets; d, doublet.

## DISCUSSION

The results of this study corroborate the ethnopharmacological information on the antimicrobial potential of *A. sativum* L. against group B Streptococcus (GBS). Although the antimicrobial actions of this plant species are well known, there is scant literature reporting its action against the pathogen (S. agalactiae) responsible for neonatal sepsis, which occurs due to vertical transmission from mother to fetus before or during childbirth (28, 29).

In addition to furnishing pharmacological evidence confirming the antimicrobial activity against S. agalactiae strains, this investigation allowed identification of the active principles present in *A. sativum* L. Although the compounds isolated in the study have previously been described in phytochemical studies, the relationship between these compounds and their actions against S. agalactiae are presented for the first time in this work (10, 30).

Garlic contains two main biologically active substances: (i) organosulfur compounds like allicin, alliin, and ajoene and (ii) nonorganosulfur compounds (31). Allicin is the main organosulfur compound present in crushed garlic and exerts numerous biological actions, such as antimicrobial (32), anti-inflammatory (33), and antitumoral (34) effects.

The compounds with antimicrobial activity found in the present study are Ƴ-glutamyl-*S*-allyl-cysteine (fraction 18), Ƴ-glutamyl-phenylalanine (fraction 20), and *E*- and *Z*-ajoenes (fraction 42). Identification of these compounds was made possible using mass spectrometry (MS) and nuclear magnetic resonance (NMR) for the conformational and structural elucidation of the small molecules found in our study.

Ƴ-glutamyl-*S*-allyl-cysteine (fraction 18) is an organosulfur peptide present predominantly in whole garlic and undergoes hydrolysis and oxidation via *S*-allyl-l-cysteine (SAC) to form alliin. When garlic is crushed, alliin is transformed into allicin through the action of the alliinase enzyme (21, 35).

Amagase (36) concluded that Ƴ-glutamyl-*S*-allyl-cysteine is converted into *S*-allyl-l-cysteine (SAC) through an enzyme reaction with Ƴ-glutamyl-transpeptidase when garlic is extracted in an aqueous solution. Yeh et al. (37) also isolated and identified several organosulfur compounds, including Ƴ-glutamyl-*S*-allyl-cysteine (GSAC), Ƴ-glutamyl-*S*-methylcysteine (GSMC), and Ƴ-glutamyl-*S*-propylcysteine (GSPC).

A study conducted by Zhu et al. (30) identified four organosulfur compounds present in garlic using the mass spectrometry method. One of these compounds was Ƴ-glutamyl-*S*-allyl-cysteine, which was also identified in the present study as fraction 18. The mass spectrum obtained for this compound by the cited authors is the same spectrum obtained in the present study.

There is a dearth of knowledge on the therapeutic effects of Ƴ-glutamyl-*S*-allyl-cysteine, but it is known to promote alliin stores in whole garlic and can also be found in processed garlic (35).

Fraction 20 was identified as Ƴ-glutamyl-phenylalanine, a peptide previously cited in the studies of Yoo et al. (10). However, in a review of the literature, no other studies involving this compound or reporting its biological actions were identified. Therefore, the current study is the first to report the antimicrobial action of this compound against S. agalactiae.

In the present study, fraction 42 proved the most effective fraction for antimicrobial action against S. agalactiae, compared to fractions 18 and 20. This fraction was identified as an ajoene (E and Z) mixture, comprised of organosulfur compounds that are formed by the degradation of pure allicin. This fraction is more stable than allicin. There are numerous studies on ajoene, including those describing actions such as antitumoral (34), apoptotic (38), antifungal (39, 40), antibacterial (24, 41), and antiplatelet (42) activity. The activity-structure relationships of ajoene have also been established for antitumoral, antifungal, and antithrombotic actions (43), but no studies reporting the antibacterial activity of the ajoene mixture (fraction 42) against S. agalactiae bacterium were found.

Using the MIC value determined for S. agalactiae ATCC 12386, the fractions with antimicrobial activity inhibited the growth of all the clinical isolates tested and the bacterial growth curve using 3× the MIC, confirming the antimicrobial activity of these fractions.

Cutler et al. (28) presented a study about the antimicrobial activity of allicin, a compound also found in processed garlic. This study differs from ours in relation to the number of strains used to determine the MIC and the compound used against the S. agalactiae strains. Comparing the MIC of the allicin (values ranging from 35 to 95 mg/liter) with the MIC of the fractions identified in our study (Table 1), the allicin was more efficient against the strains of S. agalactiae. However, our findings are also important, as we have presented new compounds with antibacterial activity against this S. agalactiae strain that have not previously been described in the literature and are more stable than allicin (34).

Hossain et al. (44) conducted a study to evaluate the antifungal activity of five isolated compounds from Eremophila alternifolia against eight yeast and two mold species. This study is like ours in relation to the method used to determine the MIC of the compounds (in triplicate); for this reason, it was used as a preliminary screening technique for antimicrobial activity.

Penicillin G is the first antibiotic of choice for intrapartum prophylaxis in pregnant women, followed by first-generation ampicillin and cephalosporin. In cases of patients allergic to penicillin G, options include clindamycin, erythromycin, and vancomycin (45). Velázquez et al. (46) analyzed 96 strains of S. agalactiae, all of which were sensitive to penicillin G at an MIC ranging from 0.012 to 0.094 μg/ml, results corroborated by the current study findings. Kaminska et al. (25) analyzed 165 strains of clinical isolates of S. agalactiae and all were sensitive to penicillin G, with an MIC ranging from 0.032 to 0.125 μg/ml. These studies are corroborated by the findings of the present work, which observed an MIC of 0.038 μg/ml.

The present study results showed that the antimicrobial effect of penicillin G was superior to that of the fractions identified. However, we believe it is important to expand the armamentarium of treatment options against GBS, particularly in cases of pregnant women allergic to penicillin G and given that IAP is effectively a prophylactic as opposed to a treatment. Moreover, the global scenario of antibacterial resistance has been of great concern for public health, and natural products can be a source of new substances to help us grapple with this problem. However, further preclinical trials assessing the pharmacodynamic, pharmacokinetic, and toxicological aspects of these substances are required to provide a basis for full clinical trials. These research efforts can pave the way for use of *A. sativum* L. or its isolated active principles, for example in a topical cream for vaginal use as a monotherapy or in association with antibiotics as a complementary therapy.

New compounds isolated from garlic with antimicrobial activity, in addition to allicin, were also discovered. This work will continue with respect to these molecules (commercially) to expand the activity spectrum, as well as for testing with a large number of clinical isolates of S. agalactiae.

## CONCLUSIONS

This study found that two peptides and an ajoene (E and Z) mixture were the most efficient compounds isolated from *A. sativum* L. (garlic) with respect to antimicrobial activity against S. agalactiae. These are extremely important findings, as they contribute to the possible development of a new medication for the topical treatment of S. agalactiae infection during pregnancy, instead of using prophylactic prevention methods during childbirth. The future development of such a treatment could help to avoid the indiscriminate use of antibiotics and the associated growth in bacterial resistance.

## MATERIALS AND METHODS

### Plant material.

The study was carried out in the Department of Physiological Sciences, Santa Casa de São Paulo School of Medical Sciences, São Paulo, SP, Brazil, and the Laboratory for Applied Toxinology at the Butantan Institute, São Paulo, SP, Brazil.

Garlic bulbs (*A. sativum* L.; Liliaceae) of the purple-striped variety were purchased in Campos Altos, MG, Brazil from the Fazenda Tri “S” Farm, situated at an altitude of 1.132 m, in November 2017. Samples of the bulbs were cultivated, and the whole plant was subsequently provided to experts at the Botanical Institute of São Paulo, who confirmed its identity. A voucher specimen, no. 502077, was deposited at the Maria Eneyda P. Kaufmann Fidalgo (SP) Herbarium, São Paulo, Brazil.

### Crude garlic extract.

Damage- and fungi-free fresh bulbs (200 g) of *A. sativum* L. were carefully peeled, washed under running water, and placed in a miniprocessor (Cadence, Balneário Piçarras, SC, Brazil) for approximately 1 min. The processed material was filtered through a no. 20 mesh sieve. The liquid thus obtained was freeze-dried (Thermo Super Modulo Pirani 501), stored in a freezer (–20°C), and labeled as crude garlic extract (CGE).

### Solid phase extraction.

CGE (2 g) was incubated in 5 ml acetic acid (2M) with magnetic stirring in a refrigerated system (ice bath) for 30 min and then centrifuged at 14,680 × *g* for 5 min; the supernatant (4.0 ml) was injected into Sep-Pak C_18_ cartridges (Waters Corp., Milford, MA, USA) equilibrated in 0.05% trifluoroacetic acid (TFA). The sample was eluted in acetonitrile (ACN; concentration, 80%), then concentrated and labeled as Sep-Pak 80% (SP80) (47).

### Bio-guided fractionation by RP-UFLC-UV.

The bio-guided fractionation of SP80 was performed using a reverse-phase ultrafast liquid chromatography with UV (RP-UFLC-UV) system on a Prominence chromatograph (Shimazdu Corp., Kyoto, Japan) with a preparative reverse-phase (RP) column Shim-pack PREP-ODS (250 mml × 50 mm inside diameter [i.d.], 15 μm). The SP80 was reconstituted in 6 ml acidified water (TFA 0.05%) and divided into three runs. Purification of the sample was carried out using a gradient of 0% to 80% ACN at a flow rate of 2.0 ml/min for 120 min. UV absorbance of the effluent was monitored at 225 nm. All eluted peaks were manually collected, vacuum-dried to completely remove the solvent, reconstituted in ultrapure water (250 μl), and stirred by vortexing and ultrasound for complete dissolution before being used in the antimicrobial activity assays (47).

### LC-DAD-MS/MS/ESI^+^ and NMR.

Mass spectrometry analysis was performed on a Shimadzu model CBM-20A chromatograph, equipped with Shimadzu LC-20AD pumps, a Shimadzu SPD-20A detector, Shimadzu CTO-20A oven, Shimadzu SIL 20AC autoinjector, and Bruker Daltonics maXis 3G mass spectrometer (Bruker Corp., Billerica, MA, USA), with a 4500-V capillary, 2-bar nebulizers, at 200°C, and a gas flow of 8 liter/min^−1^ operating in MS and MS/MS data acquisition mode with the electron spray source in positive mode (ESI^+^). For the liquid chromatography, a reverse-phase C_18_ (Phenomenex Luna) plus column (250 mm × 4.6 mm × 5 μm) was used, with a flow rate of 1.0 ml.min^−1^. The elution system used a gradient system which consisted of a mixture of (i) acidified water (TFA 0.05%) and (ii) acetonitrile (ACN) for 10 to 70 min, reaching 80% ACN (48).

Nuclear magnetic resonance (NMR) was performed using a Bruker III 500-MHz spectrometer in deuterated water (D_2_O), and the results were analyzed by unidimensional (1D) ^1^H NMR and two-dimensional (2D) heteronuclear single quantum coherence (HSQC) and heteronuclear multiple-bond correlation (HMBC) (48).

### Culture of Streptococcus agalactiae.

S. agalactiae culture (1 CFU) was grown in 4 ml of tryptic soy broth (TSB) for 18 h at 37°C overnight. After the medium reached the desired turbidity, 500 μl was transferred into 10 ml TSB and maintained until the exponential growth phase. Bacterial suspensions at 10^4^ CFU/ml were prepared by adjusting the optical density to OD_595_ = 0.250 *±* 0.005 (49).

### Liquid growth inhibition test.

The antimicrobial assay was performed against S. agalactiae ATCC 12386 using a TSB medium. Antimicrobial activity was determined using microliter broth dilution assays in 96-well sterile plates. The dried fractions were dissolved in 250 μl ultrapure water; then, 20 μl was aliquoted into each well with 80 μl microbial dilution at a final volume of 100 μl. Sterile water and TSB were used as the negative control, and penicillin G (Inlab Confiança, São Paulo, Brazil), the antibiotic of choice in the treatment of S. agalactiae infections, was used as the positive control. After 24 h of incubation at 35 ± 2°C, the growth inhibition was determined by measuring absorbance at 595 nm using a Victor3 1420 instrument (Perkin Elmer).

Five strains of clinical isolates of S. agalactiae, kindly supplied by the Salomão Zoppi Clinical Laboratory (São Paulo, Brazil) from its library, were used in the preliminary screening of the action of the fractions with antimicrobial activity (49).

### MIC.

To determine the MIC, the samples, dissolved in ultrapure water, were used to perform serial dilutions in 96-well sterile plates at a final volume of 100 μl against S. agalactiae ATCC 12386. For this, 20 μl of the fraction was applied to each well at a serial dilution of 2-fold microtiter broth dilution and added to 80 μl of microbial dilution. The MIC was considered to be the lowest concentration of each sample after 24 h of incubation at 35 ± 2°C that completely inhibited growth of the microorganism. The tests were performed in triplicate, and the average of three readings was considered (49).

### Bacterial growth curve kinetics.

To evaluate the kinetics of the antimicrobial effects of fractions 18, 20, and 42, the growth curves of S. agalactiae ATCC 12386 were determined. Bacteria were plated in 96-well plates containing TSB medium at 1 × 10^5^ CFU/well, and 20 μl of the fraction was then applied to each well at concentrations of 3× MIC with 80 μl microbial dilution at a final volume of 100 μl, respectively. The OD value at 595 nm was determined every 1 h (up to 18 h) using a Victor3 1420 instrument (Perkin Elmer). The TSB medium was used as the negative control and penicillin G as the positive control. The experiment was performed in triplicate. After an 18-h incubation, 50 μl of each fraction was plated onto blood agar plates to count the CFU (50).
